# Acute aortic dissection presenting as painless paraplegia: a case report

**DOI:** 10.1186/s13256-016-0881-z

**Published:** 2016-04-05

**Authors:** Olfa Hdiji, Nouha Bouzidi, Mariem Damak, Chokri Mhiri

**Affiliations:** Department of Neurology, Habib Bourguiba Hospital, CP: 3029 Sfax, Tunisia

**Keywords:** Aortic dissection, Painless, Paraplegia

## Abstract

**Background:**

Acute aortic dissection is an extreme emergency that is generally manifested by violent chest pain irradiating to a patient’s back and abdomen. Paraplegia due to spinal cord ischemia and infarction as a presenting manifestation of aortic dissection has been found in 2 to 5 % of patients. However, painless paraplegia is exceedingly rare and limited to a few case reports in the literature. We describe a new case with this unusual presentation of aortic dissection and here we emphasize that this condition must be considered in all patients with painless paraplegia.

**Case presentation:**

A 70-year-old Arab man with no previous known medical or surgical conditions was hospitalized for brutal heaviness of his lower limbs associated to urinary retention. A neurological examination revealed flaccid paraplegia without sensory disorder. His blood pressure and his pulse were in normal ranges. He was afebrile. His peripheral pulses were not checked. Laboratory investigations eliminated multiple organ failure. Spinal magnetic resonance imaging realized in emergency was normal. He had a cardiopulmonary arrest 1 day after his hospitalization. His autopsy report concluded a type A aortic dissection with an intimal tear at his aortic isthmus with intrapericardial rupture and extension to his intercostal and lumbar arteries.

**Conclusions:**

Acute aortic dissection is an extreme emergency that can lead to death unless there is an early diagnosis. It must be considered in any patient with paraplegia even painless. Clinical examination has a major role to play in diagnosing this condition. Apart from the neurological examination, palpation of peripheral pulses and blood pressure measurements in all four limbs is of paramount importance. Then further investigations must be carried out consisting of aortic angiography by computed tomography or by magnetic resonance imaging.

## Background

Acute aortic dissections are the most common catastrophic event affecting the aorta with an estimated annual incidence of approximately 5 to 30 per million [1]. It is divided into two classifications: type A and type B. Type A dissections involve the ascending thoracic aorta and type B dissections involve the descending thoracic aorta. Aortic dissection has a wide range of presentations. It typically presents as a sudden painful ripping sensation in the chest or back [2]. Dissection may compress or occlude a branch of the aorta and produce acute ischemia. Ischemia occurs in the arms or legs in 20 % of patients, in the kidney in 15 %, myocardium in 10 %, in the brain in 5 %, and in the mesentery or in the spinal cord in 3 % [3]. Complicating the diagnosis, 17 to 40 % of patients can present significant neurologic symptoms [4] including paraplegia which is a rare neurological manifestation found in 2 to 5 % of patients [5]. However, painless paraplegia is exceedingly rare and limited to a few case reports in the literature [6–12]. We describe a new case of acute aortic dissection presenting as painless paraplegia and here we insist that this condition must be considered in all patients with painless paraplegia.

## Case presentation

A 70-year-old Arab man presented to our department because of a sudden inability to walk. He had been well until 1 hour before admission, when he noticed the sudden onset of weakness of his legs with urinary retention. He had no chest, back or leg pain. He had no previous known medical or surgical conditions. His blood pressure was 120/56 mmHg and his pulse was 90 beats/minute. He was afebrile. He had no audible murmurs or rubs. His lungs were clear to auscultation and percussion; however, his peripheral pulses were not checked. Deep tendon reflexes were absent in his legs and he had flaccid paraplegia with no sensory disorder. Distended bladder was also noted. Laboratory investigations eliminated multiple organ failure. Acute low cervical spinal cord compression was initially suspected but spinal magnetic resonance imaging realized in emergency was normal. His clinical condition rapidly worsened 1 day after his admission. He appeared pale, diaphoretic and in extreme distress. His blood pressure was 50 mmHg systolic with a pulse of 130. Despite resuscitation, his blood pressure continued to deteriorate and he experienced cardiopulmonary arrest. Resuscitation was attempted. However, he could not be resuscitated. An autopsy revealed a type A aortic dissection with subsequent separation of the intima and media from the adventitia involving his ascending thoracic aorta with antegrade and retrograde extension. In fact, cardiac tamponade had occurred as the result of intrapericardial rupture. His intercostal and lumbar arteries were affected.

## Conclusions

Acute aortic dissection is a catastrophic illness with variable manifestations. It usually manifests as a sudden tearing chest pain radiating to the back [1]. The sudden paraplegia as in our patient can result from interruption of blood flow to the spinal cord especially to crucial zones such as the lower thoracic and lumbar segments and then ischemia of the spinal cord [7]. Aortic dissection revealed by paraplegia is rare [5] and painless acute aortic dissection in which paraplegia is the only presenting symptom is even rarer [6–12]. This unusual presentation can lead to misdiagnosis of aortic dissection with fatal evolution as in our case. Dissection of the aorta carries a very poor prognosis unless it is treated immediately. Its true incidence is generally underestimated and evidence of dissection is found in one of 400 necropsies [13]. So it is very important to differentiate this condition from other spinal vascular pathologic phenomena that produce paraplegia and are painless and to verify the existence of pulseless femoral arteries [9]. In fact, reversal of paraplegia with surgical intervention [9] as well as conservative management [14] has been described.

## Consent

Written informed consent was obtained from the patient’s next-of-kin (his son) for publication of this case report and any accompanying images. A copy of the written consent is available for review by the Editor-in-Chief of this journal.
